# Global research trends on inflammatory bowel diseases and colorectal cancer: A bibliometric and visualized study from 2012 to 2021

**DOI:** 10.3389/fonc.2022.943294

**Published:** 2022-11-29

**Authors:** Shuai Xiong, Ke Liu, Fei Yang, Yuanwei Dong, Hongcai Zhang, Pengning Wu, Yu Zhou, Lu Zhang, Qin Wu, Xiaojing Zhao, Wei Li, Lingling Yuan, Biao Huang, Rensong Yue, Li Feng, Jing Chen, Yi Zhang

**Affiliations:** ^1^ Chengdu University of Traditional Chinese Medicine, Chengdu, China; ^2^ Hospital of Chengdu University of Traditional Chinese Medicine, Chengdu, China; ^3^ XinDu Hospital of Traditional Chinese Medicine, Chengdu, China; ^4^ Anyue County People’s Hospital, Ziyang, China; ^5^ Affiliated Hospital of Jiangxi University of Traditional Chinese Medicine, Nanchang, China

**Keywords:** inflammatory bowel diseases, colorectal cancer, gut microbiome, global research trends, bibliometric

## Abstract

Inflammatory bowel disease (IBD) is a chronic non-specific inflammatory disease of intestinal tract and a common digestive system disease. Current studies have shown that IBD significantly increases the incidence of colorectal cancer (CRC), and is positively correlated with the degree and extent of inflammation of IBD. The relationship between IBD and CRC has attracted extensive attention. However, the relationship between IBD and CRC has not been systematically studied by bibliometrics and visual analysis. This study conducted bibliometric analysis based on 3528 publications from the Core Collection of Web of Science to determine the research status, research hotspots and frontiers of this field. The results show that the number of publications has increased significantly over the past 10 years. The cooperative network analysis shows that the United States, Mayo Clin and Bo Shen are the country, institution and author with the most publications respectively. Belgium, Icahn Sch Med Mt Sinai and Erik Mooiweer are the most collaborative country, institution and author respectively. Analysis of keywords and references showed that inflammation, intestinal flora, and obesity were hot topics in this field. Analysis of keyword outbreaks shows that the gut microbiome and metabolism will be an emerging new research area and a potential hot spot for future research. This study is the first to visually examine the association between IBD and CRC using bibliometrics and visual analysis, and to predict potential future research trends.

## Introduction

Inflammatory bowel disease (IBD) is a chronic autoimmune disease affecting the gastrointestinal tract (GI). It includes Crohn’s disease (CD) and ulcerative colitis (UC), characterized by repeated bouts of mucosal inflammation (1, 2). In long-standing extensive ulcerative colitis, the severity of colonic inflammation is an important determinant of the risk of colorectal neoplasia (3, 4). Longstanding UC correlates with an increased risk of developing colitis associated cancer(CAC)through cumulative inflammatory burden (5), especially when they are male, have generalized colitis and are young when diagnosed with UC (6). Based on long-term epidemiological data, IBD patients are at high risk for colorectal cancer (CRC), especially in patients with extensive UC (4). IBD-CRC is one of the major and serious complications of IBD, and although only 1% to 2% of patients with IBD develop IBD-CRC, it is responsible for approximately 15% of IBD-related mortality (7).

Many common factors influence the onset and progression of IBD and CRC, including intestinal microbiome dysregulation, changes in interleukin pathways, and tumor necrosis factor. On the other hand, the patient’s age, race, genetics, family history, dietary composition, obesity and vitamin and mineral levels also contribute to the progression (8). These multiple factors contribute to the higher incidence of CRC in IBD patients.

However, recent population-based studies have shown that with advances in CRC screening and surveillance and improved IBD treatment, the incidence of CRC in patients with UC and CD has declined (9, 10). The 3-year post-colonoscopy colorectal cancer (PCCRC) rate in IBD patients is 24.3%, which is much higher than 7.5% in the general population. This means that of all IBD patients diagnosed with CRC, 24.3 percent had a colonoscopy and had a chance to diagnose or prevent CRC at an early stage (11). The relationship between UC and CAC has influenced the development of clinical practice guidelines, with increased endoscopic surveillance recommended among UC patients starting 8 years after initial UC diagnosis. These recommendations have led to successful reductions in CAC morbidity and mortality (12).

Among the many potential factors known to increase the risk of IBD-CRC, perhaps chronic persistent inflammation is the most important (4). Therefore, prevention of inflammation may be a necessary measure to reduce IBD-CRC in clinical practice. According to the “common ground hypothesis”, microbial dysbiosis and intestinal barrier impairment are at the core of the chronic inflammatory process associated with IBD-CRC. The persistent inflammation in the colon results in increased proliferation of cells necessary for repair but this also increases the risk of dysplastic changes due to chromosomal and microsatellite instability (4). Although inflammation has been identified as a risk factor for the development of UC into CRC, the specific mechanisms need to be further investigated. The characterization of key microbial communities and their influence on the pathogenesis of UC and CAC may provide opportunities to modulate intestinal inflammation through microbial-targeted therapy. Despite recent advances in sequencing technology, further research is needed to elucidate the causal role of the gut microbiome in regulating UC inflammation (1).

Publications have demonstrated the link between IBD and CRC. However, as far as we know, no scholar has systematically analyzed the topic of this field through bibliometric analysis. Therefore, this study aims to comprehensively analyze the status and development trend of IBD and CRC from 2012 to 2021 through bibliometrics. Previous reviews only rely on individuals to study through literature review and extraction, which cannot fully reflect the spatial and temporal distribution of researchers, institutions and countries. In addition, it is difficult to visualize the internal structure of the knowledge base and research focus, and systematic, comprehensive and visual studies are rarely found.

Bibliometrics refers to the application of mathematical and statistical methods to objectively analyze the nature of the spatial distribution of scientific literature in a certain period in a specific field (13). Bibliometrics can evaluate research trends qualitatively and quantitatively according to the characteristics of literature database and bibliometrics. It can not only help scholars keep track of trends in a particular research field, but also evaluate the contributions of journals, institutions and countries in a particular research field. In addition, for the medical field, it can provide a basis for the development of clinical guidelines (14). This paper aims to discuss specific key areas that IBD and CRC may be related to future research, summarize the research status in this field, grasp the research direction and hot spots, and provide some reference for future research direction.

## Materials and methods

### Data sources

**Figure 1:**
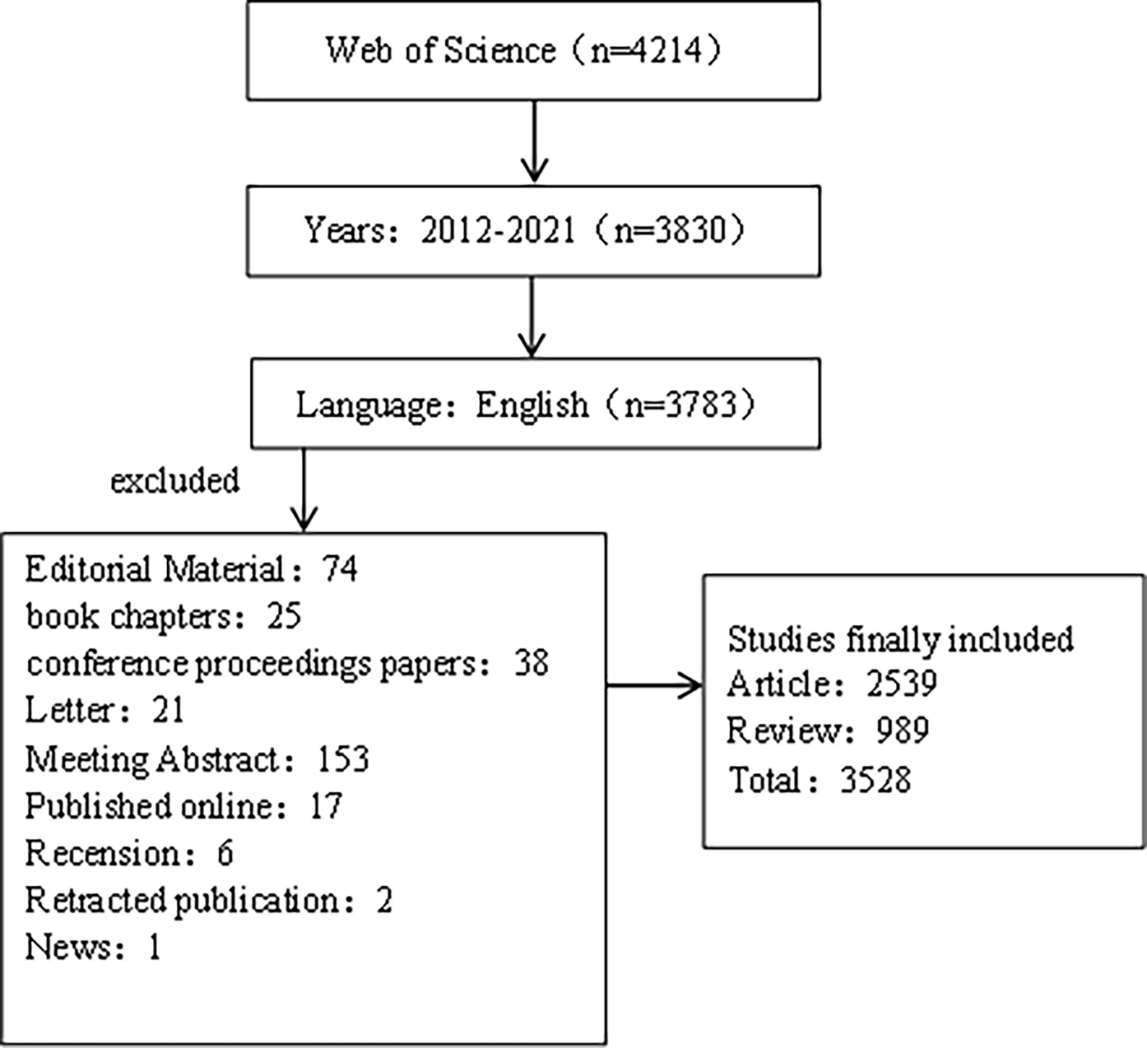
Flowchart for including and excluding literature studies.

We selected the Web of Science Core Collection (WOSCC) from Clarivate Analytics as the retrieval database for this study. WOSCC is a highly authoritative citation index database, which is widely used in scientific research and bibliometrics research at present. We use the combination of subject words and free words for line search. The subject words include: inflammatory bowel diseases and colorectal neoplasms. Free words mainly include:inflammatory bowel disease, colorectal neoplasms, colorectal neoplasm, colorectal tumors, colorectal cancer, colorectal carcinoma. We set the search language as “English”. The search document type is set to “Article or Review”. We set the publication date condition of the retrieved literature as “2011-01-01 to 2021-12-31”. The retrieval process was carried out independently by two researchers and the retrieval date was May 5, 2022. Detailed retrieval results are shown in **Figure 1**.

### Data analysis

The software used for data analysis in this study are Microsoft Office Excel 2021, Citespace.5.8.R3 and Vosviewer1.6.17. Among them, Microsoft Office Excel 2021 is used for the statistics of literature years and the production of related tables; Citespace.5.8.R3 is used for the analysis of the number of published countries, institutions and authors in the data and the cooperation relationship, and the production of keyword co-occurrence map, keyword burst map. Vosviewer1.6.17 is used to analyze highly cited and co-cited references in the data. The analysis in this study uses WOSCC to analyze the publication output, web of science subject category, publication year, author and other functions. After that, the selected documents were imported into Cite Space, and time span was 2010–2020. Select the node based on the type of analysis need to perform. The specific parameter settings in Cite Space are as follows: Time slicing: January 2012–December 2021, Term source: Title, Abstract, Author Keywords and Keyword Plus, Node type: Author, Institution, Country and Keyword. Link strength: Cosine. Selection Criteria: Top N =50. In the generated map, the color of the circular nodes represents the time when the article was published. The thickness of the node is positively related to the frequency. the thickness of the line describes the strength between the projects, and the color of the line describes the year when the two projects first collaborated. The nodes in the map represent elements such as author, country/region, or institution. The link lines between the nodes indicate the collaboration relationship. The larger the circle, the more articles published. The wider the line, the stronger the relationship. The outermost purple ring represents the Centrality. The centrality value represents the cooperation intensity, and the higher the value, the stronger the cooperation. Use VOS viewer software to statistically analyze highly cited and co-cited documents. First, import the results obtained through WOSs into the software. Then set number of terms to be selected:10. Finally, import the results to excel for sorting.

## Results

### Literature search results

We retrieved a total of 4214 relevant literatures through the web of Science Core Collection (WOSCC) database, and 3528 were screened. The specific screening process is shown in **Figure 1**.

### Publication years

**Figure 2:**
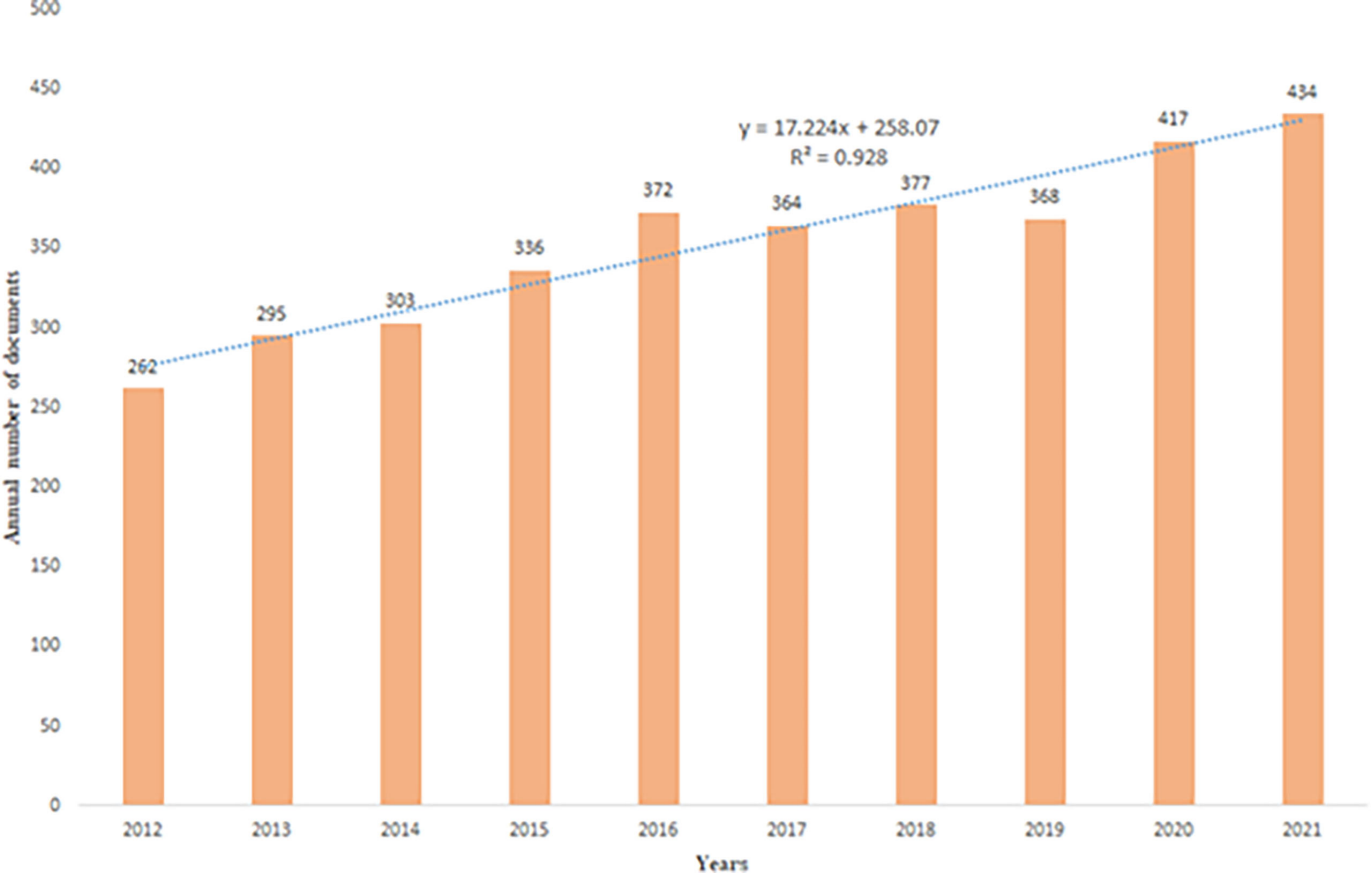
The number of publications by year and the overall trend.

As shown in **Figure 2**, the number of articles published in 2012 was at least 262, and the number of articles published in 2021 was at most 434. From 2012 to 2021, the number of articles published in joint studies on IBD and CRC showed a linear growth trend, satisfying the functional relationship “Y = 17.224x + 258.07 R² = 0.928”.

### Analysis of publication volume and cooperative relationship

**Figure 3:**
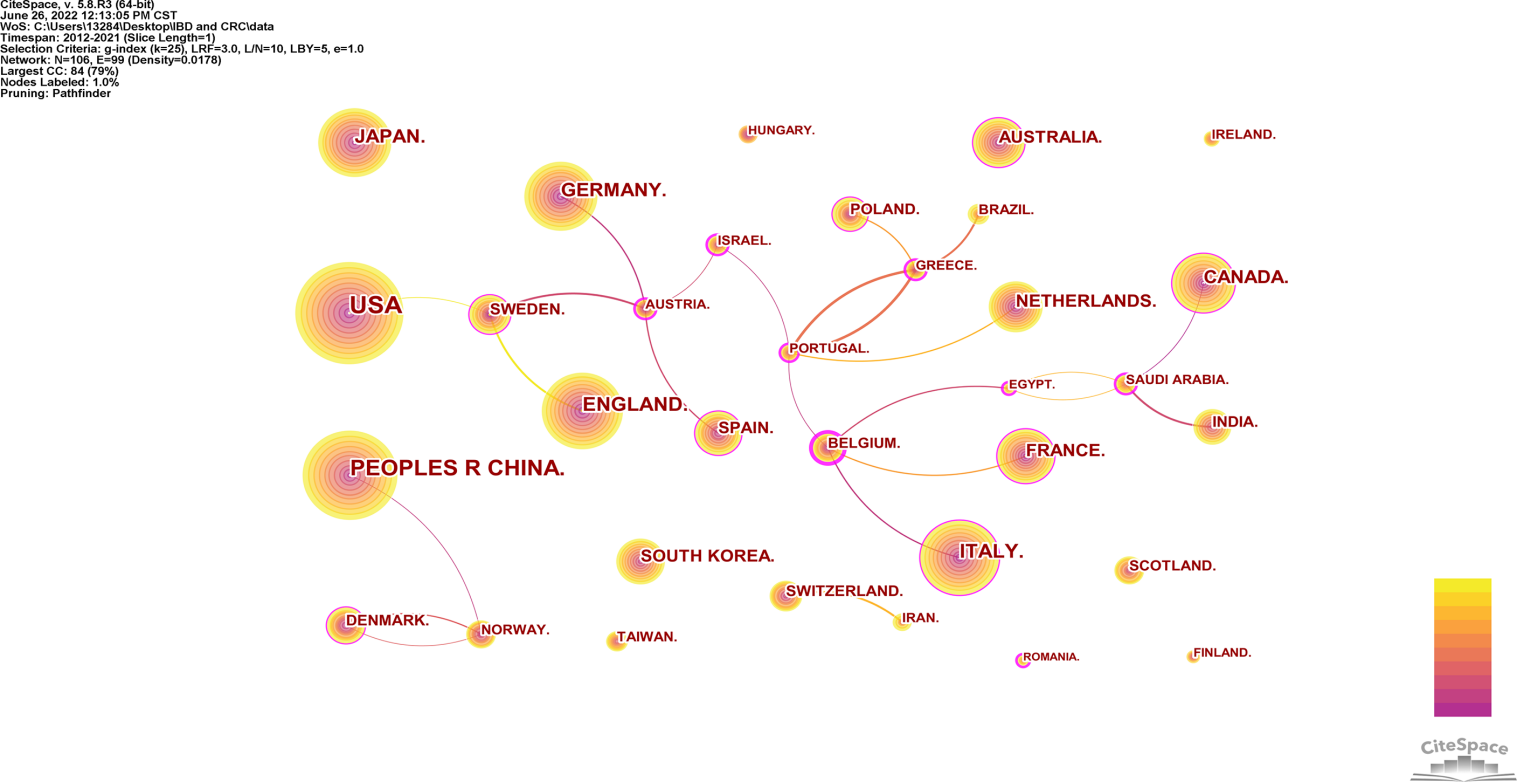
Visualization of cooperative relations of countries with more than 20 articles.

A total of 106 countries/regions published papers on IBD and CRC. The top 10 countries/regions in terms of publication volume and centrality are shown in **Table 1**. The top 10 countries published 3,261 papers, accounting for 92.43% of the total. The United States had the largest number of articles with 1,110, accounting for 34% of the top 10 countries, followed by China (550, 17%) and Italy (275, 8%). However, the national centrality of the top ten publications is not high. In contrast, Belgium, Saudi Arabia and the United Arab Emirates have high centrality, but they post very little. Countries/regions with more than 20 articles are visualized in **Figure 3**. It can be seen that the United States cooperated more with Switzerland, Italy and France cooperated more with Belgium.

**Table 1:** Top 10 countries/regions, institutions, and authors in terms of publications and centrality.

Item		Publications		Centrality	
	Rink	Name	Number	Name	Number
**country/region**	1	USA	1110	BELGIUM.	0.45
	2	PEOPLES R CHINA.	550	SAUDI ARABIA.	0.38
	3	ITALY.	275	U ARAB EMIRATES.	0.37
	4	ENGLAND.	268	ISRAEL.	0.35
	5	GERMANY.	235	ROMANIA.	0.35
	6	JAPAN.	227	PORTUGAL.	0.33
	7	CANADA.	178	MALAYSIA.	0.32
	8	FRANCE.	147	RUSSIA.	0.29
	9	NETHERLANDS.	140	AUSTRIA.	0.26
	10	AUSTRALIA.	131	EGYPT.	0.26
**institution**	1	Mayo Clin	78	Icahn Sch Med Mt Sinai	0.3
	2	Cleveland Clin	55	Hosp Beatriz Angelo	0.3
	3	Univ Washington	42	Karolinska Inst	0.26
	4	Univ Med Ctr Utrecht	40	Fudan Univ	0.26
	5	Massachusetts Gen Hosp	39	Columbia Univ	0.25
	6	Univ Chicago	37	Dartmouth Hitchcock Med Ctr	0.25
	7	Univ Oxford	36	NCI	0.17
	8	Johns Hopkins Univ	36	Emory Univ	0.17
	9	Shanghai Jiao Tong Univ	35	Univ Med Ctr Utrecht	0.16
	10	Univ Toronto	35	Cleveland Clin Fdn	0.15
**author**	1	Bo Shen	35	Erik Mooiweer	0.12
	2	Bas Oldenburg	25	Jeanfrederic Colombel	0.08
	3	Laurent Peyrinbiroulet	21	Bas Oldenburg	0.07
	4	Edward V	17	Antonino Spinelli	0.07
	5	Markus F Neurath	14	Bo Shen	Bo Shen
	6	Peter D Siersema	13	Edward V	0.06
	7	Gerhard Rogler	13	Andrea E Van Der Meulende Jong	0.06
	8	Evelien Dekker	12	Janindra Warusavitarne	0.04
	9	Frank Hoentjen	12	Ecco Ca	0.04
	10	Antonino Spinelli	12	Ashwin N Ananthakrishnan	0.03

**Figure 4:**
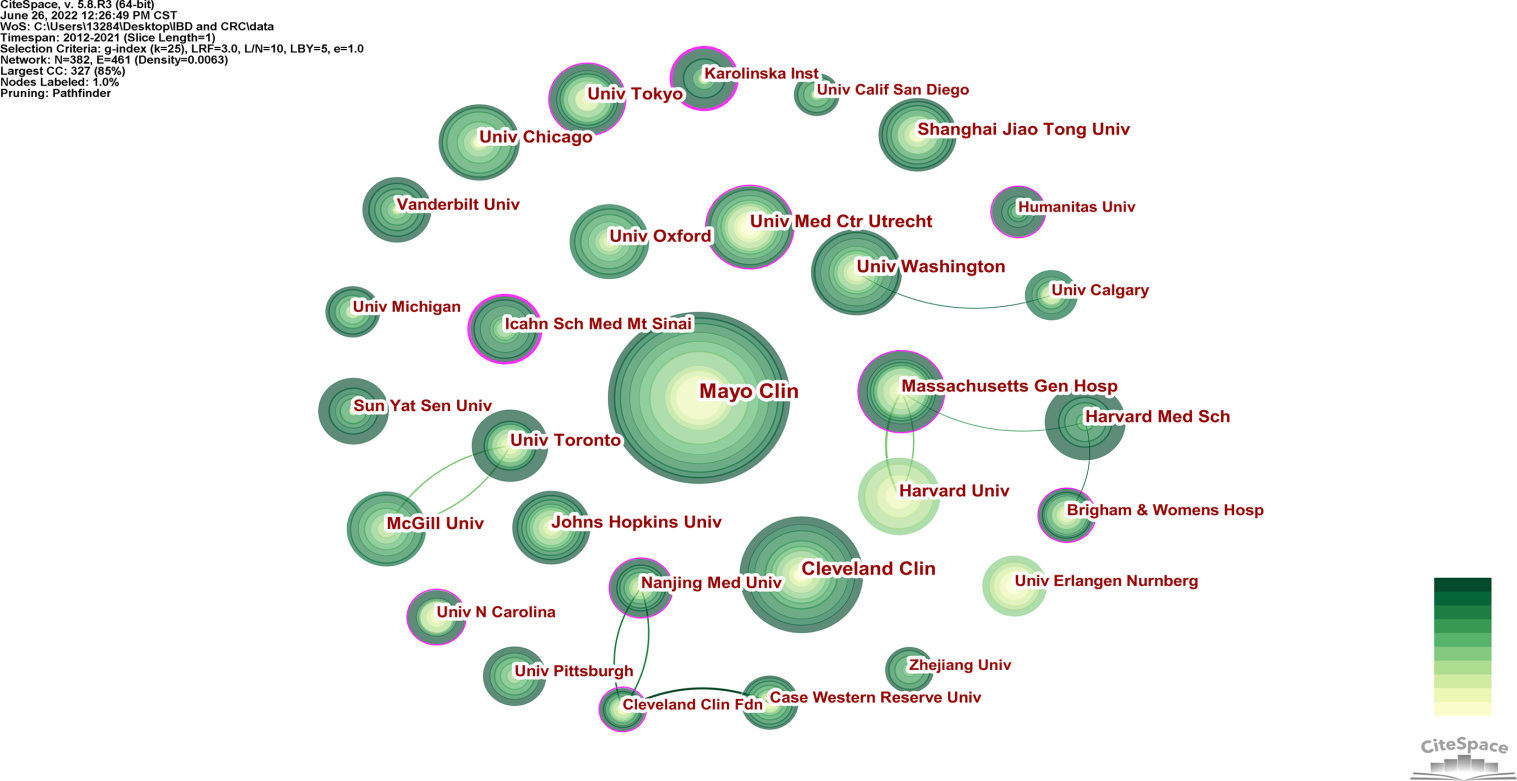
Visualization of cooperation relationship of institutions with more than 20 articles.

As shown in the **Table 1**, a total of 382 institutions published papers in this field, with Mayo Clin (78 papers) leading the way, followed by Cleveland Clin (55 papers) and Univ Washington (42 papers). The institution with the largest centrality was Icahn Sch Med Mt Sinai, followed by Hosp Beatriz Angelo and Karolinska Inst. Based on the analysis of publications and centrality, Univ Med Ctr Utrecht (Publications: 40, centrality: 0.16) and Cleveland Clin Fdn (publications: 55, centrality: 0.15) were the leading institutions in the field. According to **Figure 4**, the cooperation relationship between institutions is not very close. Mayo Clin, the largest institution in the node, has less cooperation with other institutions. On the contrary, Massachusetts Gen Hosp has many collaborations with Harvard Univ and Harvard Med Sch, and has many publications among them.

**Figure 5:**
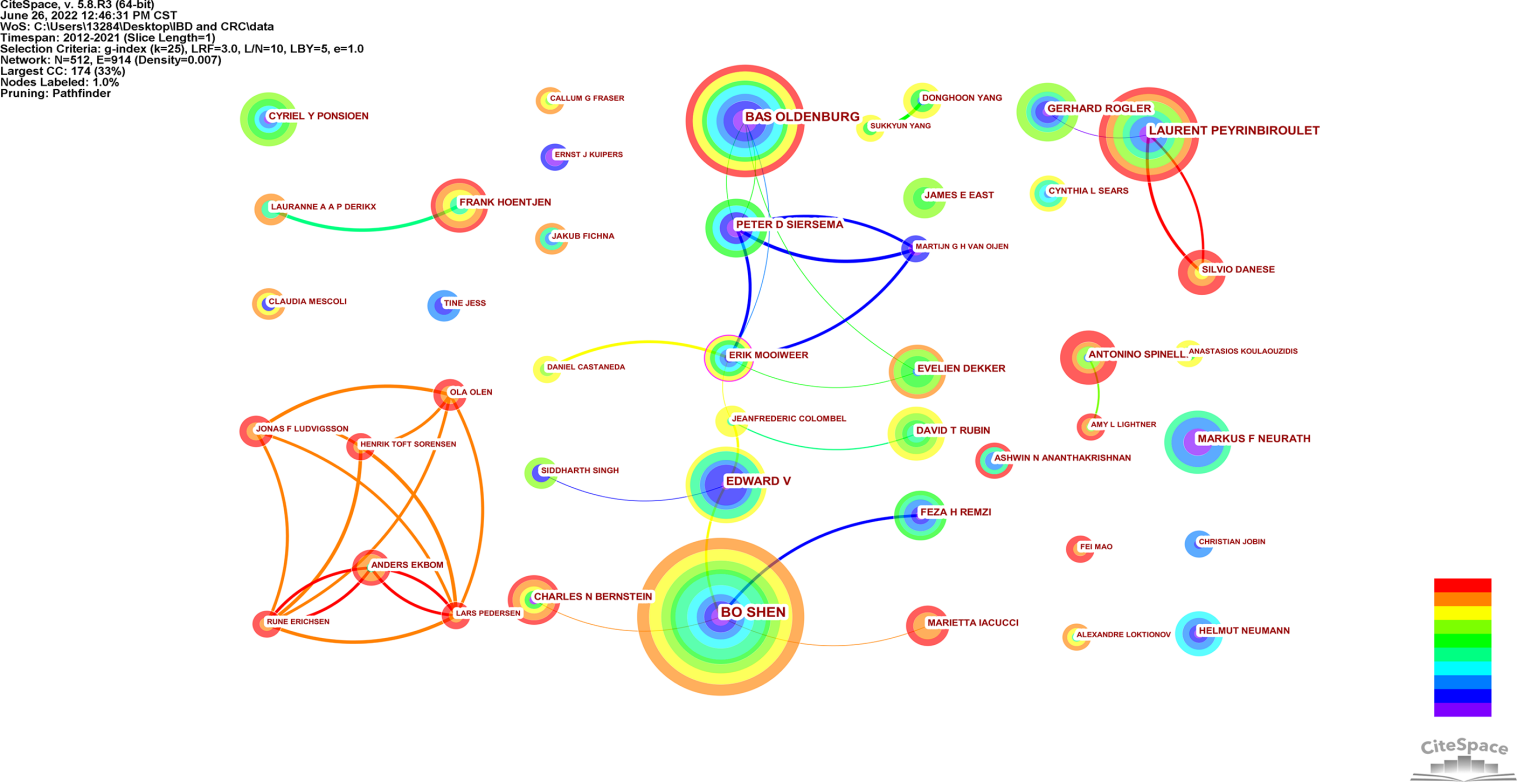
Visualization of cooperative relationship of authors with more than 5 articles.

A total of 512 authors published papers on the relationship between IBD and CRC from 2012 to 2021, and the top 10 authors in terms of publication volume and centrality are shown in **Table 1**. Three authors with more than 10 publications are Bo Shen (35 papers), Bas Oldenburg (25 papers) and Laurent Peyrinbiroulet (21 papers). The more central authors are Erik Mooiweer, followed by Jeanfrederic Colombel and Bas Oldenburg. It can be seen that Bo Shen, Bas Oldenburg, Edward V, Antonino Spinelli have an important influence on the joint research of IBD and CRC. The authors with more than 5 papers are visualized in **Figure 5**. The author with the largest node, Bo Shen, has a close cooperative relationship with Edward V, Charl Es N Bernstbn, Feza H Remzi and other authors. It is worth mentioning that in recent years, Jonas F Ludvigsson, Olaolen, Henrik Toft Sorensen, Claudiamescoli and Anders Ekbom have formed a close cooperation network.

### Analysis of co-occurring keywords, burst term and cluster analysis

**Figure 6:**
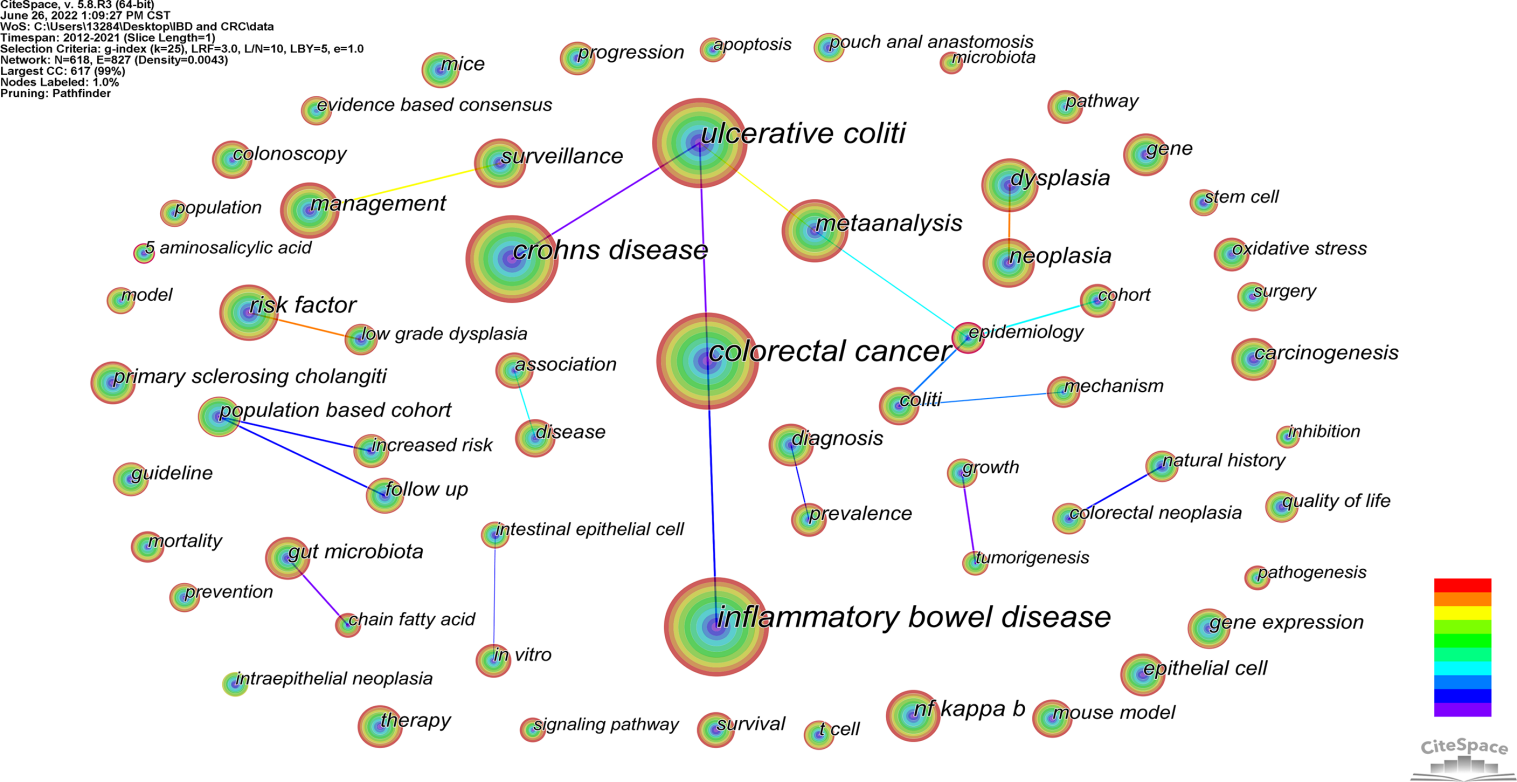
Visualization with keyword frequency more than 50.

Co-occurrence keywords can be used to analyze and discover research hotspots. There are 618 keywords in this study, and **Table 2** lists the top 20 keywords based on co-occurring frequency. **Figure 6** shows the visualization of keywords with keyword frequency more than 50. The higher the frequency, the more hotter it is. The connection between the keywords indicates that they are jointly studied. The top 4 are “inflammatory bowel disease”, “colorectal cancer”, “Ulcerative Colitis”, and “Crohn’s disease”. Obviously, the frequency of UC was significantly higher than that of CD. In addition, from 2012 to 2021, the risk factors, mechanism, diagnosis and treatment of the relationship between IBD and CRC were also studied.

**Table 2:** Top 20 keywords in terms of frequency and centrality.

Rank	Keywords	Frequency	Centrality
1	inflammatory bowel disease	1456	0.01
2	colorectal cancer	1428	0.01
3	ulcerative colitis	1066	0.03
4	Crohns disease	683	0.00
5	meta-analysis	252	0.03
6	risk factor	214	0.01
7	neoplasia	195	0.00
8	nf kappa b	194	0.01
9	dysplasia	193	0.01
10	management	182	0.03
11	surveillance	168	0.00
12	diagnosis	137	0.00
13	gut microbiota	118	0.02
14	population based cohort	116	0.07
15	carcinogenesis	112	0.01
16	epithelial cell	109	0.02
17	primary sclerosing cholangitis	105	0.01
18	gene expression	103	0.02
19	mice	102	0.03
20	therapy	100	0.00

**Figure 7:**
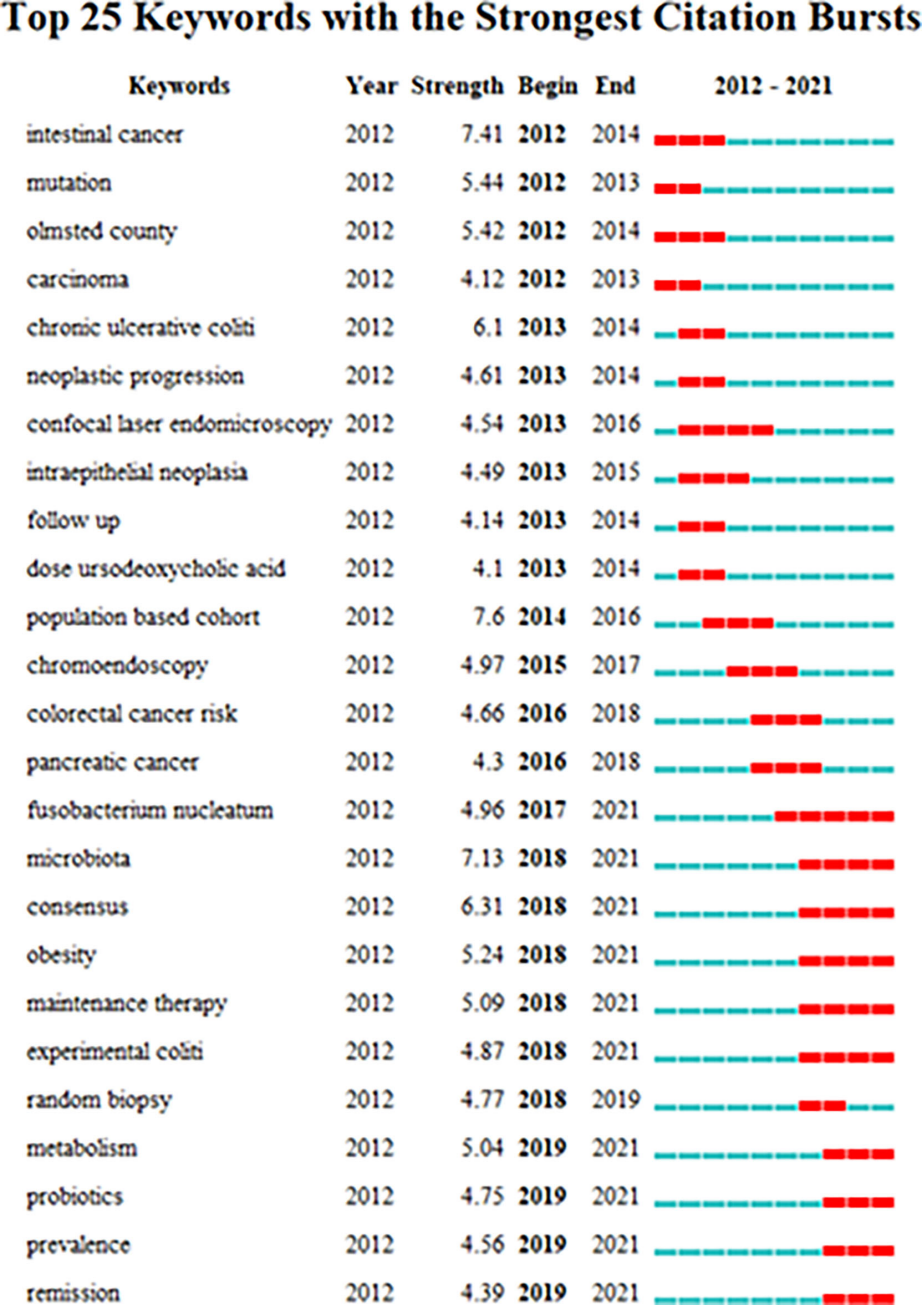
The top 25 keywords with the strongest citation bursts.

Keyword burst refers to a sudden increase in research content at a given moment, which can be used to predict the potential development trend of the research field. **Figure 7** shows 2012 - 2021 IBD and CRC common area 25 keywords from the initial research focus on “mutation”, “tumor progress”, “to oxygen dose bear bile acid”, “to the current research of clostridium” nuclear “and” bacteria “, “consensus”, “fat”, “maintenance treatment”, “experimental colitis”, “random”, “metabolism”, “probiotic” and “popular”, “task Changes in research. It can be seen that in recent years, the research focus has shifted from mechanism to diagnosis and treatment, among which fusobacterium nucleatum, Microbiota, probiotics, Obesity, and metabolism, and “maintenance therapy” may be the research trend.

Keyword clustering can clearly understand the different research directions in the research field and classify and summarize the research topics. In this study, LLR algorithm was used to cluster IBD and CRC-related keywords from 2012 to 2021. There were 21 clusters in total, and the cluster value of modules was Q=0.7888 (>0.3), clustering average contour value S=0.9213 (>0.7), indicating that the clustering effect is reasonable. The first 10 clusters are shown in **Table 3**. It can be seen that there are many studies on the mechanism of the relationship between inflammatory bowel disease and colorectal cancer comorbidity. Now more and more studies have been transferred from mechanism research to clinical research. How to reduce the transformation of IBD into CRC is the topic of current research, such as colorectal resection, activity marker monitoring, dietary intervention, intestinal flora adjustment, etc.

**Table 3:** Top 10 Keyword clustering.

Cluster	Frequency	Sihouette	Keywords
#0 double-edged sword	43	0.861	double-edged sword; novel selective jak2 inhibitor; necroptosis adaptor ripk3; critical function; tea polysaccharide
#1 dietary intervention	37	0.925	dietary intervention; recent advance; triple viable probiotics; emerging partner; allergic diseases
#2 postoperative outcome	35	0.962	postoperative outcome; laparoscopic surgical stapling device; invasive colon; rectal division; colorectal anastomotic leak rate
#3 inflammation-associated colorectal carcinogenesis	34	0.940	inflammation-associated colorectal carcinogenesis; mast cell; dioscorea batata; setd2 modulate; histone methyltransferase
#4 observational study	33	0.946	observational study; dietary fish oil; microrna-143 target; abdominal complaint; of-care fecal calprotectin
#5 activity marker	33	0.966	activity marker; clinical relapse; fecal calprotectin level; pomegranate juice;6-year prospective multicenter
#6 post-colonoscopy colorectal cancer	33	0.899	post-colonoscopy colorectal cancer; advanced colorectal adenoma; genomic characterization; cost-effectiveness analysis; novel risk stratification
#7 target nf-kappa b	32	0.913	Target; nf-kappa b; novel synthetic; gel-forming muc2 mucin; targeting common metabolic pathway; integrated informatics analysis
#8 epigenetic regulation	31	0.894	epigenetic regulation; new potential omega-3 polyunsaturated fatty acid target; m2 macrophage polarization; targeting activation-induced cytidine deaminase; persistent colonic inflammation
#9 intestinal microbiota	29	0.931	intestinal microbiota; lactase non; persistent people; dairy food consumption; adjacent normal colonic epithelium

### Analysis of highly cited references and co-cited references

Highly cited literature refers to the publications cited frequently and with a wide range of influence. As shown in **Table 4**, the two literatures “Gastrointestinal Inflammation Targets Cancer-Inducing Activity of the Microbiota” (1216 times) and “The Gut Microbiota and Host Health:A New Clinical Frontier” (1089 times) were cited for more than 1000 times, and both of them were studies on intestinal microbes, indicating that the current research hotspot is intestinal microbes.

**Table 4:** The top 10 co-cited references and highly cited references.

Items	Rank	Title	DOI	Citations
**Co-cited reference**	1	The risk of colorectal cancer in ulcerative colitis: a meta-analysis	10.1136/gut.48.4.526	671
	2	Ulcerative colitis and colorectal cancer. A population-based study.	10.1056/nejm199011013231802	282
	3	AGA technical review on the diagnosis and management of colorectal neoplasia in inflammatory bowel disease.	10.1053/j.gastro.2009.12.035	280
	4	Risk of colorectal cancer in patients with ulcerative colitis: a meta-analysis of population-based cohort studies.	10.1016/j.cgh.2012.01.010	263
	5	Severity of inflammation is a risk factor for colorectal neoplasia in ulcerative colitis.	10.1053/j.gastro.2003.11.010	250
	6	Intestinal inflammation and cancer.	10.1053/j.gastro.2011.01.057	245
	7	Inflammation and cancer IV. Colorectal cancer in inflammatory bowel disease: the role of inflammation.	10.1152/ajpgi.00079.2004	233
	8	Decreasing risk of colorectal cancer in patients with inflammatory bowel disease over 30 years.	10.1053/j.gastro.2012.04.016	228
	9	Cancer risk in patients with inflammatory bowel disease: a population-based study.	10.1002/1097-0142(20010215)91:4<854::aid-cncr1073>3.0.co;2-z	224
	10	Guidelines for colorectal cancer screening and surveillance in moderate and high risk groups (update from 2002).	10.1136/gut.2009.179804	203
**Highly cited reference**	1	Intestinal Inflammation Targets Cancer-Inducing Activity of the Microbiota	10.1126/science.1224820	1216
	2	The gut microbiota and host health: a new clinical frontier	10.1136/gutjnl-2015-309990	1089
	3	The microbiome and cancer	10.1038/nrc3610	870
	4	Ulcerative colitis	10.1016/S0140-6736(12)60150-0	821
	5	Adenoma-linked barrier defects and microbial products drive IL-23/IL-17-mediated tumor growth	10.1038/nature11465	802
	6	Structural segregation of gut microbiota between colorectal cancer patients and healthy volunteers	10.1038/ismej.2011.109	659
	7	What is the Healthy Gut Microbiota Composition? A Changing Ecosystem across Age, Environment, Diet, and Diseases	10.3390/microorganisms7010014	656
	8	Quality indicators for colonoscopy	10.1111/j.1572-0241.2006.00673.x	616
	9	Burden and Cost of Gastrointestinal, Liver, and Pancreatic Diseases in the United States: Update 2018	10.1053/j.gastro.2018.08.063	601
	10	The burden of inflammatory bowel disease in Europe	10.1016/j.crohns.2013.01.010	551

Co-cited references are the basis and basis of later research. The more times of co-citation, the greater the contribution of the literature to future research. As shown in **Table 3**, the meta-analysis published in 2001 by Eaden JA et al., which cited the most, demonstrated the relationship between UC and CRC, indicating that UC has a high risk of developing into CRC.

## Discussion

### Publication trends analysis

In recent 10 years, the research on inflammatory bowel disease and colorectal cancer has maintained a high level, and the joint research on inflammatory bowel disease and colorectal cancer has also been a hot spot in the field of digestion and tumor research.

### Cooperative relationship

It is worth noting that Italy, with a centrality of 0.15, ranks 14th, but ranks 3rd in the number of publications. This shows that Italy is not only publishing more, but also cooperating more. China ranked second in the total number of published articles, but its centrality was only 0.06, reflecting the lack of international collaborative research in China. It is expected that China will strengthen international cooperation in the study of IBD and CRC in the near future.

#### Intestinal inflammation increases the risk of inflammatory bowel disease developing into colorectal cancer

Co-cited references are the basis of subsequent research, while highly cited references reflect the research trend. According to **Table 4**, the basis of the research focuses on the following aspects:the relationship between IBD and CRC, the role of inflammation in the development of ulcerative colitis into colorectal cancer, epidemiological investigations related to IBD and CRC; and guidelines for colorectal cancer surveillance. We can find that IBD increases the risk of CRC, and inflammation plays an important role in this process, providing a basis for further mechanism studies. Studies (15) showed that the estimated overall prevalence of CRC in any UC patient was 3.7%. But in a population-based cohort, UC increased the risk of CRC by 2.4 times. The severity of colon inflammation is an important determinant of the risk of CRC (3). The longer and wider the inflammation, the greater the risk of developing CRC.

#### Fusobacterium nucleus is an important pathogenic factor of colorectal cancer

Studies have found that Clostridium, particularly Fusobacterium nucleatus(F. nucleatum), has become a potential cause of CRC susceptibility. As far as we know, F. nucleatum, a Gram-negative bacterium present in the oral cavity, is the primary causative agent of chronic periodontitis (16). It has attracted interest in the past decade because of its association with CAC. Although studies have found that it may play an important role in the progression of CRC (17), its exact role and related mechanisms in the progression of IBD-CRC remain unclear. A recent study found that F. nucleatum significantly increased the malignancy of azomethane (AOM)/DSS induced CRC. And compared with untreated CRC cells, F. nucleatum synergistically increased the invasiveness and epithelial-mesenchymal transformation (EMT) characteristics of DSS-treated mouse CRC cells. In conclusion, Clostridium nucleatus promotes the occurrence of IBD-CRC (18). It was also found that AOM/DSS-treated ctP-knockout mice had increased tumor load, suggesting that CTPS are essential for maintaining intestinal homeostasis (19). This field is in its infancy and further work is needed to determine whether bacterial and/or defensive peptides play an important role in the development of IBD-CRC.

#### Obesity is a risk factor for colorectal cancer

On the other hand, as a recognized risk factor, obesity causing about 15-20% of cancers (20). It is noteworthy that CRC is also a type of cancer closely related to obesity (21, 22). In Europe, around 11% of CRC cases have been attributed to overweight and obesity (23). Visceral fat or abdominal obesity seems to be of greater concern than subcutaneous fat obesity, and any 1 kg/m2 increase in body mass index confers more risk (24). Diet-induced obesity accelerates CAC in mice by increasing inflammation and immune cell recruitment. Dietary fat increases taurocholic acid production in the colon, leading to dilation of Bilophila wadsworthia and colitis in interleukin-10 deficient mice (25). In addition, high fat diet drives colorectal tumorigenesis through inducing gut microbial dysbiosis, metabolomic dysregulation with elevated lysophosphatidic acid, and gut barrier dysfunction in mice (26). Meanwhile, CRC has been linked to a sedentary lifestyle and obesity. Obesity might increase the likelihood of recurrence or mortality of the primary cancer and may affect initial management, including accurate staging (24). This suggests that obesity treatment strategies that reduce inflammation can be easily implemented in patients through diet and lifestyle interventions (27). But it is unclear whether bariatric surgery can help reduce the risk of CRC (24). The research on how obesity increases the risk of colorectal cancer is undoubtedly a research hotspot, but the direct link between obesity-induced dysbiosis and CRC remains to be further studied (28). Further understanding of the molecular and cellular mechanisms associated with obesity and inflammation and colorectal cancer is needed to develop potential new therapies (29).

#### The relationship between intestinal flora and IBD and CRC is the focus of research

Although inflammation has been identified as a risk factor for the progression of UC to CRC, the exact mechanism is unclear. On this basis, the latest research trends are suggested by highly cited references. Highly cited literatures suggest that the mechanism of UC developing into CRC is related to intestinal flora. Microbiome and host form a complex “superorganism”, and the two have a symbiotic relationship (28). When the environment changes (infection, diet, or lifestyle) or the microbiome changes, it can disrupt this symbiosis and promote disease. More and more evidences indicate that microbiome plays a key role in carcinogenesis (27, 30).

99% of the microbiota is located in the gastrointestinal tract, so the gastrointestinal microbiota has been studied the most (28). A large number of studies in patients and mice have linked microbiota to the occurrence of colorectal cancer (31). On the one hand, several studies have shown that gut microbiota is an essential factor in driving inflammation in the colon, and this inflammatory environment is related to CRC development (32). On the other hand, CRC patients with obesity (OB-CRC) display a specific gut microbiota profile characterized by a reduction in butyrate-producing bacteria and an overabundance of opportunistic pathogens, which in turn could be responsible, at least in part, for the higher levels of proinflammatory cytokine IL-1β, the deleterious bacterial metabolite TMAO, and gut permeability found in these patients (33). Furthermore, intestinal inflammation and genotoxin-induced DNA damage of intestinal cells have been proposed as the possible mechanisms responsible for the role of microbial dysbiosis in carcinogenesis. The enrichment of a variety of bacterial species in the intestinal tract, including Fusobacterium nucleatum, anaerobic digestion streptococcus and enterotoxic Bacteroides fragilis, has been proved to contribute to the occurrence of colorectal cancer by inducing tumor proliferation, promoting inflammation, leading to DNA damage and protecting tumors from immune attacks (34). In a word, Microbiota regulates various mechanisms of carcinogenesis, including inflammation, metabolism and genotoxicity (28). This suggests that the above aspects may provide microbiome targeted prevention strategies for cancer. Microbium-based cancer prevention strategies may be the trend of future research.

#### Adjusting intestinal flora to prevent and treat CRC has become a research hotspot

Associations between microbial dysbiosis, chronic inflammation, autoimmunity, and tumorigenesis are well established (1). Because inflammation alters the composition of gut microbes and promotes IBD to become CRC, microbiome related treatments have been extensively studied in clinical trials. Gut microbiota modulation, with the aim to reverse established microbial dysbiosis, is a novel strategy for prevention and treatment of CRC (34). In recent years, the use of probiotics to treat CRC is becoming increasingly popular, as they have achieved positive and favorable results in many *in vitro*, *in vivo* and clinical studies, and it has become a popular candidate for the prevention and treatment of CRC (35). Probiotics, such as Lachnospiraceae species, Bifidobacterium animalis and Streptococcus thermophilus, are found to be depleted in CRC patients. These bacteria are suggested to exert a protective effect against CRC (36). Probiotics, or other commensal microbiota, confer colonization resistance by competing for nutrients and adhering surface on epithelial cells or mucus (37), or alternatively by antagonizing pathogen colonization through aggregation with pathogens, so robiotic administration is suggested to restore microbial dysbiosis and maintain intestinal microbial balance by occupying host tissue and preventing colonization of pathogenic bacteria. Furthermore, probiotics can produce metabolites such as lactic and acetic acid, or bacteriocins, which inhibit pathogen growth by lowering luminal Ph (38). The mechanisms of probiotics in the treatment of CRC are mainly as follows: establishing symbiosis, improving intestinal barrier function, regulating intestinal immune system, producing anticancer compounds and degrading carcinogenic compounds in the intestinal environment (39, 40). It is worth noting that probiotics may have adverse effects on immunocompromised patients. And there has also been some safety concerns regarding probiotic use in cancer patients, including the risk of bacterial translocation and systemic invasion, as well as the potential transmission of resistant genes to resident microbiota and the rise of antimicrobial resistance (34). Therefore, inactivated probiotics can be used as a substitute for live bacteria in these patients (41). Probiotics prevention and treatment of CRC may continue to be a focus of future research due to their high safety and low adverse reactions. In addition, given the potential role of the gut microbiome in the pathogenesis of CAC, the targeted regulation of gut bacteria by prebiotics, homologous genes, antibiotics and FMT may also be a trend of research (1).

#### The relationship between metabolic syndrome and CRC has become a future research trend

Similarly, metabolic syndrome (MetS) is a group of metabolic risk factors including abdominal obesity, hypertension, hyperglycemia, and dyslipidemia (42). Prevalence of MetS ranges from 34.8% to 41.9% in the US and from 18% to 46% in Europe (14, 43). Studies on intestinal microbiome dysregulation and the resulting inflammatory state help us understand the underlying pathogenesis (44). A nested case-control study (21) found that the association between MetS and CRC was caused by abdominal obesity and abnormal glucose metabolism. A recent meta-analysis (45) also found that MetS are associated with an increased risk of CRC incidence and cancer-specific mortality, but further research is needed to clarify the mechanism. Predictably, the interaction between IBD, CRC, intestinal microflora, obesity and metabolic syndrome may be a research hotspot.

Through Cite Space and VOS viewer software, this study carried out bibliometric and visual analysis in the field of IBD and CRC, intuitively displayed the current research status of research countries/regions, institutions and authors, and provided references for scholars in related fields for further research. Through this study, it can be found that at present, intestinal flora disorder, inflammation, obesity and metabolic disorders may be important influencing factors for IBD to develop into CRC. It can be predicted that adjusting intestinal flora, controlling intestinal inflammation and regulating metabolism will become the research trend in the future. Our study may promote the development of this field and laying foundation for future research.

## Conclusion

This is the first study to assess and quantify the global research productivity associated with IBD and CRC, presenting the overall picture of the topic and exploring future research directions. Since 2012, the volume of literature on IBD and CRC has continued to grow. The US, China, Italy and England are the most productive regions. Currently, the main hot topics related to both IBD and CRC are the following: the intestinal microflora associated with the development of IBD into CRC, microbiome based cancer prevention strategies, obesity and metabolic syndrome in relation to colorectal cancer.

## Limitations

The study had some limitations. First of all, our data only come from the WOSCC and only contain literature in English, which may lead to incomplete data and certain deviations. Secondly, some authors or institutions have different name formats in the WOSCC, and the research count may be scattered. As a result, their names may not appear in the results. Finally, due to publication bias, all conclusions of this study are from published studies, but many other studies (mainly negative results) may never be published, so there may be publication bias. However, we still consider that the findings of our analysis were adequate to characterize accurately the state of IBD and CRC research at the global level.

## Data availability statement

The original contributions presented in the study are included in the article/supplementary material. Further inquiries can be directed to the corresponding authors.

## Author contributions

SX and YiZ jointly determined the theme, SX wrote the draft, and PW, LZ, HZ, FY, BH, LF and YD supplemented and revised the manuscript. KL, YuZ, QW, XZ, JC, RY, LY and WL reviewed the manuscript. All of them contributed to the manuscript and agreed to submit the final version of the manuscript.

## Funding

This research was funded by National Natural Science Foundation of China (81973821), National Natural Science Foundation of China (8227152900), The Xinglin Scholars Program (MPRC2022014), Hospital of Chengdu University of Traditional Chinese Medicine (19PJ04), Sichuan Administration of Traditional Chinese Medicine (CKY2021106), Jiangxi Provincial Natural Science Foundation Youth Fund (20202BAL216065), Jiangxi Provincial Education Department Science Program (GJJ201259), Jiangxi Provincial Science and Technology Department (20212BAG70037), Sichuan Provincial Department of Science and Technology (2021YFS0268), Sichuan Provincial Department of Science and Technology (2022JDKP0082), Sichuan Provincial Department of Finance (CJJ2022055).

## Conflict of interest

The authors declare that the research was conducted in the absence of any commercial or financial relationships that could be construed as a potential conflict of interest.

## Publisher’s note

All claims expressed in this article are solely those of the authors and do not necessarily represent those of their affiliated organizations, or those of the publisher, the editors and the reviewers. Any product that may be evaluated in this article, or claim that may be made by its manufacturer, is not guaranteed or endorsed by the publisher.
